# 3D Imaging of Nanoparticle Distribution in Biological Tissue by Laser-Induced Breakdown Spectroscopy

**DOI:** 10.1038/srep29936

**Published:** 2016-07-20

**Authors:** Y. Gimenez, B. Busser, F. Trichard, A. Kulesza, J. M. Laurent, V. Zaun, F. Lux, J. M. Benoit, G. Panczer, P. Dugourd, O. Tillement, F. Pelascini, L. Sancey, V. Motto-Ros

**Affiliations:** 1 Institut Lumière Matière, UMR5306 Université de Lyon 1 – CNRS, Université de Lyon, 69622 Villeurbanne cedex, France; 2 Andor Technology, Springvale Business Park, Belfast BT12 7AL, UK; 3 CRITT Matériaux Alsace, 67305 Schiltigheim, France

## Abstract

Nanomaterials represent a rapidly expanding area of research with huge potential for future medical applications. Nanotechnology indeed promises to revolutionize diagnostics, drug delivery, gene therapy, and many other areas of research. For any biological investigation involving nanomaterials, it is crucial to study the behavior of such nano-objects within tissues to evaluate both their efficacy and their toxicity. Here, we provide the first account of 3D label-free nanoparticle imaging at the entire-organ scale. The technology used is known as laser-induced breakdown spectroscopy (LIBS) and possesses several advantages such as speed of operation, ease of use and full compatibility with optical microscopy. We then used two different but complementary approaches to achieve 3D elemental imaging with LIBS: a volume reconstruction of a sliced organ and in-depth analysis. This proof-of-concept study demonstrates the quantitative imaging of both endogenous and exogenous elements within entire organs and paves the way for innumerable applications.

Nanoparticles (NPs) have been an attractive research topic in preclinical and medical fields both for imaging^1^,^2^,^3^,^4^ and therapeutic purposes^5^,^6^,^7^. For the latter, NPs can be used for drug/gene delivery^8^,^9^,^10^, immunotherapy^11^,^12^, or radiosensitization^13^,^14^,^15^. The applicability of nanomaterials is largely governed by their size. While large-size NPs are generally used for delivery purposes, recent studies note that smaller NPs (<12 nm) are better candidates for tumor targeting. These small NPs may indeed have better accumulation and penetration in tumors and also present less toxicity because they are preferentially eliminated through the kidneys^16^,^17^,^18^. However, the characterization of these pharmacologically attractive candidates within biological organs remains highly challenging, particularly because of their small size. Any modification applied to such small NPs (for example, fluorescence labeling) may modify their shape, size, and/or charge and therefore affect their biodistribution^19^. Moreover, the label may escape or detach from the NP. Consequently, nanomaterials dedicated to *in vivo* applications must be thoroughly evaluated. NPs must be tailored to their intended application and a broad monitoring of every stage of pharmacokinetics (absorption, distribution, metabolism, and excretion) is crucial for determining the biological activity and toxicity of NPs. These processes require the use of imaging approaches, ideally with 3D capabilities^4^,^20^.

Only a few techniques allow the study of the biodistribution of nanomaterials in biological tissues and in 3D. Among them, optical imaging may be the most widely used. This technique is applicable to any NP from the subcellular to the whole-body scale and has a typical resolution of 200 nm^20^,^21^. However, in this case, nanomaterials must be tagged with dyes. NP labeling is also generally required for nuclear imaging, such as positron emission tomography (PET). The relatively low resolution (typically 1.2 mm) of PET is adapted for studies at the whole-body scale^4^. Magnetic resonance imaging (MRI) can be used to follow NPs containing elements with nuclear spin, such as ultrasmall superparamagnetic iron oxide (USPIO) particles or gadolinium (Gd) chelates^22^,^23^. However, similar to computed tomography (CT)^24^, MRI cannot discriminate the tissue from the contrast agent, which limits its role to enhancing contrast dynamics. Except in very specific cases, elemental imaging is the only way to conduct investigations using label-free, *i.e.*, modification-free, metal-based nanoparticles.

Among the elemental imaging approaches such as transmission electron microscopy combined with energy dispersive X-ray analysis (TEM-EDX)^21^,^25^, synchrotron radiation microanalysis (SXRF)^26^,^27^,^28^, and laser ablation inductively coupled plasma mass spectrometry (LA-ICP-MS)^29^,^30^,^31^, TEM-EDX is the only technique that achieves sub-nm resolution, thus allowing the direct visualization of a single NP. All the other techniques probe areas or volumes that are generally much larger than the NP size. However, these techniques can successfully image the biodistribution of NPs, especially when the elements constituting the particles are different from the elements of the tissue itself^32^,^33^,^34^,^35^,^36^. These scanning techniques could generate 3D images through a “layer by layer” analysis, but their relatively slow acquisition rate (generally limited to ∼1 Hz/pixel) strongly limits the analysis of large samples and their application to 3D elemental imaging.

We recently developed an alternative elemental imaging technique based on laser-induced breakdown spectroscopy (LIBS) to image the distribution of different NPs containing Gd, calcium (Ca), or gold (Au) injected into rodents^37^,^38^,^39^,^40^. LIBS is a technique with a large range of applications^41^,^42^ and is generally attractive when field deployment is required^43^,^44^. By applying LIBS to biological imaging, we recently demonstrated the possibility of imaging elements at the organ scale with ppm-scale sensitivity and a pixel size of up to 10 × 10 μm^2 ^ ref. 37. Although LIBS is not as sensitive as LA-ICP-MS (<500 ppb) or as high-resolution as μXRF (<1 μm), LIBS has the advantage of a fast operating speed and an all-optical tabletop instrument that is compatible with standard optical microscopy. The scanning speed can be up to 100 times faster than other techniques, allowing 3D investigations to be conducted on large biological samples within reasonable time periods. Here, we demonstrate for the first time that LIBS imaging can be used for the 3D imaging of biological organs. This methodological proof-of-concept study was conducted in the context of the renal clearance of theranostic Gd-based NPs in rodents. These NPs - named AGuIX - have a size of less than 5 nm and are developed for image-guided radiotherapy^13^,^38^. We present different methodologies for 3D imaging across different length scales, from the global NP distribution within the entire organ to specific regions of interest with higher resolution. Our results demonstrate that LIBS is suitable for label-free, NP 3D imaging of biological tissue and represents a promising and powerful approach for preclinical investigations.

## Insights into LIBS biological imaging

**Figure 1:**
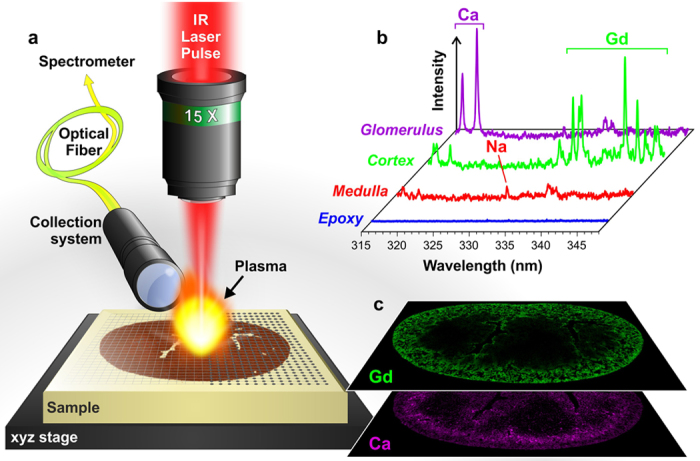
General protocol for LIBS imaging. (**a**) Schematic view of the LIBS instrument showing the major components: the microscope objective used to focus the laser pulse, the motorized platform supporting the sample and the optical detection system connected to the spectrometer via an optical fiber. (**b**) Example of single-shot emission spectra covering the 315–345 nm spectral range recorded in different regions of the mouse kidney with the characteristic emission lines of calcium (Ca), sodium (Na), and Gd. (**c**) Example of relative-abundance images of Gd (green) and Ca (violet) represented in a false color scale.

In LIBS, the laser-induced plasma generated by focusing laser pulses on the surface of the sample of interest allows a specific optical response to be elicited from the elements constituting the sample^41^,^42^. This specific response, resulting from the electronic relaxation of atoms and ions excited by the high plasma temperature, is collected and analyzed using an optical spectrometer. The elemental “signal” (atomic and ionic emission lines) is then extracted from the recorded spectrum, and elemental maps can be obtained in a pixel-by-pixel manner by scanning the sample surface over the region of interest (Fig. 1).

The implementation of LIBS is simple because a single laser pulse can simultaneously sample the material by laser ablation and excite the vaporized elements by heating the plasma plume. Hence, the acquisition speed for LIBS is mainly governed by the laser frequency rate. The apparent simplicity of the setup endows LIBS with a series of advantages over other elemental imaging methods; these benefits include an all-optical design, operation in ambient atmosphere, and fast acquisition. Apart from the instrumentation, this technique has also great assets regarding its analytical potential, such as the lack of restrictions on the number of elements that can be simultaneously detected assuming elemental lines within the probed spectral range. LIBS is especially efficient for metals, which often have intense emission lines in the UV-visible range, although organic elements can also be detected. It also has quantitative capabilities, either through the use of standards with known concentrations^37^ or specific multi-calibration methods, as described below.

With LIBS, the accessible imaging performances (spatial resolution and sensitivity) are inextricably linked to the laser ablation process. The resolution is ultimately governed by the size of the ablation craters, whereas the sensitivity largely relies on the amount of vaporized material and also on the excitation capability of the laser pulse^37^. In addition, laser ablation is a violent process because it is accompanied by different mechanisms, such as shock wave formation and thermal diffusion through the sample. These effects might cause much more sample deterioration than caused by the ablation itself and might make it difficult to analyze thin tissue slices (∼10 μm). All these points emphasize that application of LIBS to tissue imaging mainly depends on the optimization of the laser/biological matter interaction. Performance can be improved to an extent by optimizing irradiation parameters (wavelength, energy density, pulse duration, and beam profile quality). Nevertheless, hardening of the samples is always preferable to improve the mechanical stability of the sample and, therefore, the ablation shot-to-shot repeatability.

## Results

### From 2D to 3D LIBS imaging

**Figure 2:**
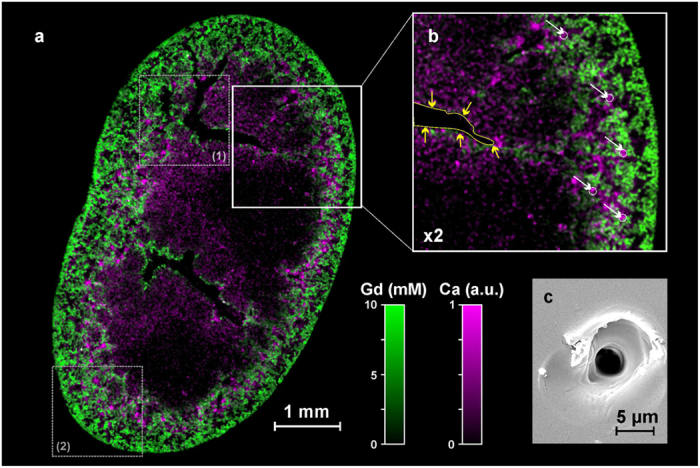
High-definition 2D elemental image. (**a**) Quantitative Gd (green) and relative Ca (violet) biodistributions in a coronal murine kidney section, 3 hours after Gd-based NP administration (500 × 720 pixels, spatial resolution of 10 μm). The Gd concentration is expressed in millimoles per liter (mM). The gray squares (1) and (2) indicate the specific regions analyzed by in depth imaging ablation (see below). (**b**) 2x magnification of the square region presented in (**a**). The yellow arrows indicate the edge of a large collecting duct, and the white arrows indicate renal corpuscles. (**c**) Example of a single-shot crater observed in scanning electron microscopy.

To date, the best LIBS imaging performances have been obtained after fixing and embedding the biological samples in epoxy resin, which is a typical protocol for electron microscopy experiments. This process allows the tissue to be embedded in a hard form with minimal modifications of the biological structure^45^. Besides, although the distribution of metals can be rather heterogeneous inside the tissue, their low concentrations (generally lower than ∼0.1%) allow the sample to be considered as a unique matrix. In other words, matrix effect from one position to another can be neglected. In our experiments, infrared nanosecond laser pulses of 500 μJ were focused by a 15x magnification objective. A typical 2D distribution of Gd and Ca within a coronal murine kidney section is presented in Fig. 2a,b. The ablation craters are ∼5 μm, and this image was obtained with a lateral resolution of 10 μm (Fig. 2c). From a biological point of view, this image clearly showed NP accumulation (*i.e.*, Gd) in the cortex (peripheral region of the kidney) and in specific zones of the medulla (central region). In the cortex, NPs are heterogeneously distributed with zones containing either high or low concentrations of NPs. In the medulla, NPs are only observed in regions surrounding the collecting ducts (shown by yellow arrows in Fig. 2b). In addition to monitoring NPs, it might be interesting to follow the elements that naturally exist in the organ, especially if they provide information about its anatomical structure. As an example, Ca is a constitutive element of the tissue that is more homogeneously distributed in the organ; however, it is more concentrated in the renal corpuscles (shown by white arrows, Fig. 2b).

When attempting to conduct 3D LIBS imaging, it is important to find an equilibrium between the resolution achieved in the 3 spatial dimensions and the time needed to obtain the information from a sample of a given size. We present two complementary methodologies that allow the 3D elemental analysis of murine kidneys containing Gd-NPs. The first strategy was to slice an epoxy-embedded kidney into consecutive ∼200 μm-thick sections to study the global distribution of NPs throughout the entire organ. This experiment allowed several sections of the kidney to be mapped and assembled in a stack and then modeled as a volume. The second 3D imaging strategy exploited the invasive nature of LIBS with repeated 2D analysis of the same renal regions. This in-depth elemental imaging process enabled the progressive analysis of a volume with a resolution of ∼10 μm in all dimensions.

### 3D imaging at the organ scale: slicing the sample

**Figure 3:**
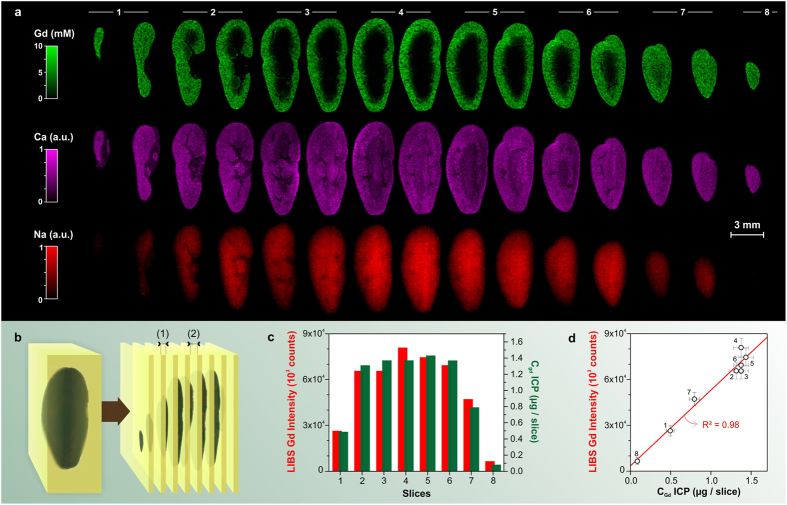
Global 3D NP distribution and quantification. (**a**) Series of elemental images of Gd, Ca and Na obtained on each side of 8 adjacent coronal sections. The kidney was sampled 3 hours after the administration of Gd-based nanoparticles. (**b**) Sample embedded in epoxy resin and subsequent slicing. Distances (1) and (2) are both equal to 200 μm and respectively correspond to the width of the organ slices and of the cutting-off saw (wasted material), (**c**) Slice-by-slice comparison between the LIBS Gd intensity and Gd mass determined by ICP-OES. (**d**) LIBS intensity calibration.

The epoxy-embedded kidney was sliced using a high-precision cut-off machine. On average, 8 slices were obtained from a mouse kidney sample cut in coronal planes. Each slice was 200 μm thick (±5 μm) and was separated from the following slice by 200 μm (±10 μm) (Fig. 3b). We performed a 30,000-measurement LIBS mapping (120 × 250) sequence with a lateral resolution of 35 μm on both sides of each slice. The time required to analyze one map (*i.e.*, one side of each slice) was less than 1 hour at a 10 Hz laser repetition rate. Elemental images of Gd, Ca, and Na were finally obtained for all the analyzed faces, constituting a total of 15 consecutive sections (Fig. 3a).

The quantification of Gd was performed using a new method developed specifically for LIBS imaging. Each slice was analyzed independently by inductively coupled plasma optical emission spectrometry (ICP-OES) after the LIBS measurements. These analyses provided the total mass of Gd in each section. This Gd mass was then compared slice by slice to the LIBS Gd intensity, which was obtained by summing all the pixel intensities on both sides of each slice (Fig. 3c). The observed good agreement suggested that the small volume of material analyzed by LIBS was sufficiently representative of the entire slice volume. In addition, the important variation in Gd mass along the different slices allows a calibration curve to be built (Fig. 3d). Once the volume of biological tissue contained in each slice was determined (Supplementary Fig. 1), the relative-abundance image of Gd was subsequently transformed into a quantitative-abundance image, expressed in millimoles per liter (mM) (Fig. 3a). This calibration yielded an extrapolated relative limit of detection (LoD) for Gd of 15 ppm for a single-shot measurement. This limit of detection (LoD) was estimated as 3 times the noise, estimated from the spectrum baseline, divided by the slope of the analytical curve, and considering a resin density 1.2 kg/L. Finally, the global Gd concentration was determined to be 3.2 mM in the entire kidney. This value was in perfect agreement with previous studies performed in similar experimental conditions^38^.

**Figure 4:**
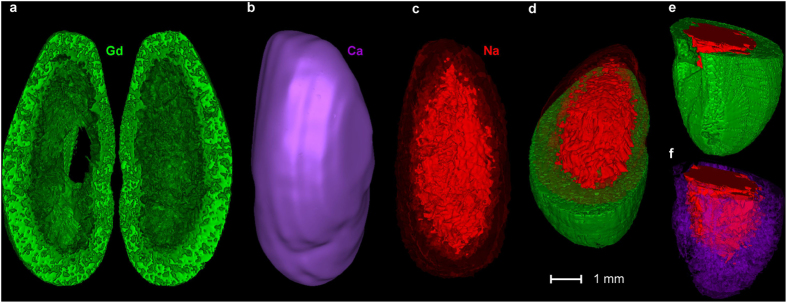
3D imaging at the entire-organ scale. Examples of elemental 3D representations, *i.e.*, Ca (violet), Na (red), and Gd (green). (**a**) 3D coronal visualization of the Gd in the kidney. Light green shows the high Gd concentrations. (**b**) Distribution of Ca approximates the global volume of the organ. (**c**) Distribution of Na allows the visualization of the contrast between the low cortical Na concentrations and the elevated medullar concentrations. (**d**–**f**) Sections of the kidney allowing the observation of the combination of Gd with Na on the coronal or axial sections (**d**,**e**) or with Ca (**f**).

The series of consecutive elemental images were then stacked into 3D images. Complete 3D models covering the entire organ volume were then constructed for Gd, Na, and Ca. With these models, cross-sectional views can be redrawn at any depth and/or orientation. Examples of 3D representations are illustrated in Fig. 4 for Gd, Ca, and Na. The Gd distribution clearly showed the NP accumulation in the cortex (Fig. 4a). The homogeneous distribution of Ca allowed volumetric information regarding the sample to be collected (Fig. 4b). Sodium was more concentrated in the medulla (Fig. 4c), which might reflect its physiological, active reabsorption in this region. The combination of different elemental distributions allowed visualization of the NP distribution during the physiological renal elimination of the NPs (Fig. 4d–f). The high cortical Gd concentrations contrasted with the high concentrations of Na predominantly in the medulla (Fig. 4d,e).

### 3D imaging by in-depth ablation

The in-depth ablation process was performed by repeating the laser shots over the same area of interest. As mentioned previously, this layer-by-layer mode provided several consecutive 2D sequences and allowed a higher resolution (∼10 μm) to be achieved in all 3 dimensions. In this LIBS 3D-imaging strategy, the minimal accessible depth resolution depends on the amount of material removed, which is correlated with both the laser pulse properties and the material itself. The depth resolution might be affected by the properties of the resin used for sample embedding, and in general, the best resolutions are achieved with the hardest samples. Moreover, the sample surface must be adequately refreshed layer after layer, by operating in an abrasion-like regime. With our laser pulse properties (500 μJ, 1064 nm, 5 ns), this regime was obtained by setting the lateral resolution at 12 μm.

**Figure 5:**
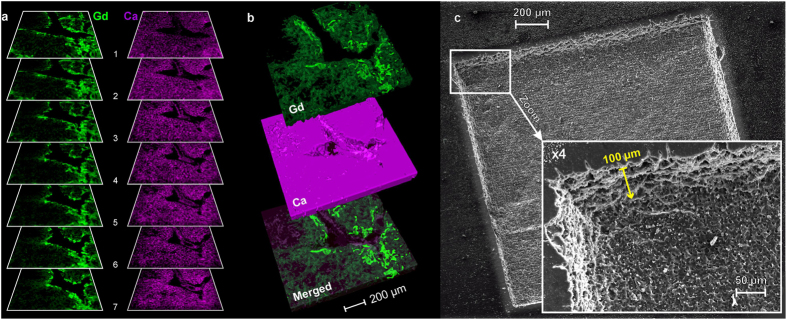
3D depth profile imaging: collecting ducts. (**a**) Successive in-depth images of Gd (green) and Ca (violet) in an ∼2 mm^2^ region located in the medulla (lateral resolution of 12 μm and depth resolution of 15 μm). (**b**) Example of 3D representations of Gd and Ca and a dual-color overlay for Gd/Na. (**c**) Wide-angle and magnified images of the sample surface recorded via SEM after the completion of the seven successive 2D LIBS scans. The total ablated depth indicated by the yellow arrow was 100 μm.

We performed 7 consecutive scans (Fig. 5a) in an area centered on a collecting duct system (square #1 in Fig. 2a). Every single 2D sequence yielded a 120 × 120 pixel image. The laser focus was adjusted before every additional layer with an incremental value of 15 μm, *i.e.*, the depth resolution. 3D models were then constructed for both Gd and Ca. The 3D renderings for Gd, Ca and an overlay of both elements are shown in Fig. 5b. The NPs appeared to be trapped in the urothelium, *i.e.*, the epithelial cells from the collecting duct. The sample surface was observed with scanning electron microscopy (SEM) (Fig. 5c). SEM images showed that the material was removed uniformly over the entire scanned area, layer after layer. The expected and measured total depth of this experiment was 100 μm. This value was measured at different positions of the sample using an optical microscope. This ablation mode induced the creation of rectilinear edges around the ablated surface, creating artefactual side effects with strong signal degradation visible in the close-to-the-edges pixels. Consequently, we removed the 5 external pixels of each line and row during the analysis to construct the 3D model (Fig. 5a,b).

**Figure 6:**
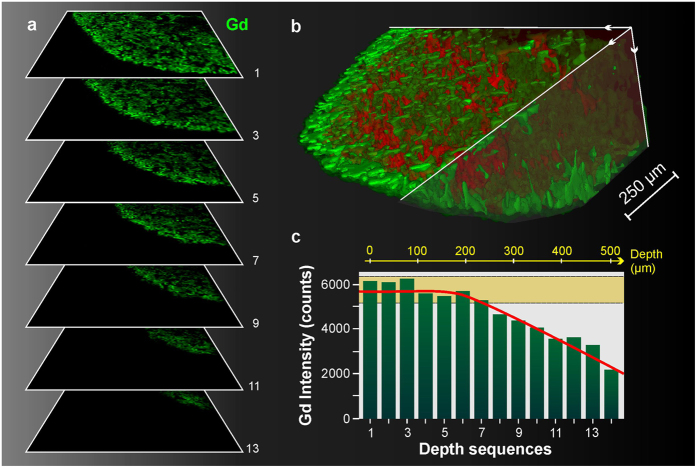
3D depth profile imaging: cortex. (**a**) Successive images of Gd (green) in an ∼2 mm^2^ region located in the cortex (lateral resolution of 12 μm and depth resolution of 35 μm). Seven images are presented from a total of 14. (**b**) Example of a 3D representation of both Gd and Na (red) for the ∼500 μm total depth analysis. (**c**) Evolution of the Gd intensity, determined using the 5% of pixels with the highest Gd intensity, as a function of the probed layer.

We performed another analysis from a peripheral zone of the kidney, in the region delimited by square #2 in Fig. 2a. In this case, 14 consecutive scans were performed with the same experimental and sequencing parameters (120 × 120 pixels with a 12 μm step). To determine whether the properties of the resin would influence the analysis, we used a different embedding resin containing less hardener. In this case, we observed an increased amount of removed material per laser pulse, which was estimated to be an approximately 35 μm depth per layer. The 2D images of 7 from the 14 consecutive acquisitions are shown for Gd in Fig. 6a, and a 3D rendering for an overlay of Gd and Na is also presented in Fig. 6b. This experiment demonstrated the possibility of analyzing a surface deep within the sample (*i.e.*, 500 μm depth); however, global signal deterioration occurred after a depth of 200 μm (Fig. 6c). This finding might be explained by a loss in the signal collection due to the geometry of our detection system. In this particular case, the signal degradation was compensated using a normalization by the level of the spectrum background. The 3D observation shown in Fig. 6b allowed an observation of the natural tortuous architecture of the cortex. The strong heterogeneity of NPs in the cortical architecture is clearly noticeable. The regions containing high and low levels of Gd are thought to be the proximal and distal tubules, respectively.

## Discussion

We developed an innovative method with 3D capability to image NPs in tissue at the organ scale and with a cellular-level degree of information (∼10 μm). The proposed label-free, quantitative, and multi-elemental approach has no equivalent because of its speed of operation, its ease of use, and its full compatibility with optical microscopy. Two 3D strategies were detailed. The first strategy consisted of slicing the biological sample into consecutive sections. This process allows the global distribution of nanoparticles to be studied in the entire organ volume. The second strategy exploits the laser ablation properties to gradually obtain the depth-profile of the sample by conducting repeated 2D sequences of the same area. In this in-depth imaging method, specific zones of the sample were observed with a cellular scale resolution (∼10 μm). The two strategies are highly complementary because they provide information about the NP distribution at different scales and with a reasonable time of analysis. Although the methodology was demonstrated for Gd-based NPs on a model-type sample, *i.e.*, a kidney, there is no restriction on using this technology for tissues with more complex architectures (such as the brain, lungs or tumors) or for any type of metal-based NP (gold, silver, platinum, and others). This methodology has the potential to become a valuable tool in preclinical investigations, in particular for studying the elimination, toxicity, and pharmacokinetics of nanomaterials, as well as for any biological or medical application involving metallic ions. In addition, the proposed calibration method was demonstrated for Gd. It can be extended to any other elements as long as the volume of material analyzed by LIBS is sufficiently representative of the slice volume. This strategy avoids the use of external calibrators, which solves the issue of finding or developing specific reference samples.

The invasive nature of LIBS imaging undeniably restricts its use on preclinical animal samples or on human biopsies. The use of epoxy prevents complementary immunohistological analysis of tissues. Accordingly, we are currently working on the optimization of the technique for the analysis of paraffin-embedded samples. Efforts are being made to integrate Raman and fluorescence imaging systems to extend the optical capability of our bio-LIBS instrument. For specific applications requiring higher sensitivity, the use of a second laser pulse that is tuned to specifically excite the element of interest would significantly improve its performance in terms of sensitivity. In addition, although 10–100 Hz acquisition rates have already been achieved, companies are currently attempting to improve the technology by developing both Gaussian lasers with a high frequency rate and high-speed detectors. These future improvements would allow an increase in the LIBS imaging speed to the KHz scale. The ideal development of a fully automated sample sectioning, measurement, and fast analysis setup would also bring the bio-LIBS technology to the leading edge of 3D elemental imaging of biological tissue.

## Methods

### Sample preparation

The animal experiments were approved by the local ethics committee (CECCAPP, agreement #LS-2013-004). All animal procedures are in accordance with the French Government’s Guidelines. Briefly, female NMRI nude mice (7 weeks’ old, Janvier, Le Genest-Saint-Isle, France) were anesthetized with isoflurane (4% initiation, 2% maintenance). Gd-based NPs (8 mg of AGuIX) were IV injected in anesthetized mice. Kidneys were sampled at the indicated times after injection and were embedded in epoxy. After sampling, each kidney was perfused and fixed with 2% glutaraldehyde in 0.1 M sodium cacodylate buffer (pH of 7.4) overnight. The samples were then rinsed three times for 10 minutes each in 0.1 M sodium cacodylate buffer and dehydrated in a series of ethanol solutions of increasing concentration, ending with propylene oxide. The samples were then embedded in EPON using a mix of EMBed-812/DDSA/NMA (5:4:2). DMP-30 was used as a hardener (2% of the mix volume). For Fig. 6, the proportion of these reagents was (5:6:1). The surface of the sample was prepared using a high-precision cut-off machine (Accutom 50 from Struers, Champigny sur Marne, France). The sections were 200 μm thick and separated by 200 μm.

### Experimental setup and data acquisition

The instrumental setup was based on a homemade optical microscope. The LIBS experiment used Nd:YAG laser pulses of 1064 nm, focused onto the sample by a 15x magnification objective (LMM-15X-P01, Thorlabs). The pulse duration was 5 ns, and the repetition rate was 10 Hz. During the experiments, the sample could be translated along the 3 axes by an XYZ motorized stage with a travel range of 50 mm in each direction. The measurements were performed at room temperature and under ambient conditions. During the sample scan, trigonometric surface positioning was used to compensate for any flatness anomalies and to accurately control the objective-to-sample distance. A beam shutter was used to control the delivery of the laser pulse to the sample such that only one plasma plume was produced for each position of the sample. The light emitted by the plume was collected by a quartz lens and focused onto the entrance of a round-to-linear fiber bundle composed of 19 fibers with a 200-μm core diameter. With this collection system, the observed surface was equal to the whole optical fiber diameter (∼1 mm) and allows light emission from the all the plasma volume to be collected. The output of the fiber bundle was connected to a Czerny-Turner spectrometer equipped with a 1200 l/mm grating blazed at 300 nm and an intensified charge-coupled device (ICCD) camera (Shamrock 303 and iStar, Andor Technology, respectively). The ICCD camera was synchronized with the Q switch of the laser, and the spectrum was acquired with a delay of 500 ns and a gate of 3000 μs. The width of the entrance slit of the spectrometer was set to 30 μm. In this configuration, a spectral measurement range of 30 nm was possible with a spectral resolution of approximately 0.15 nm. To perform the mapping experiments at the greatest possible speed, the movement of the sample was synchronized with the opening of the beam shutter, and the spatial resolution was set by adjusting the speed of the translation stage. The laser energy was stabilized throughout the experiment by using a servo control loop to improve the long-term stability of the laser output. This loop was achieved by using a power meter and a computer-controlled attenuator (ATT1064, Quantum Composers). Homemade software developed in the LabVIEW environment controlled the entire system and allowed the performance of automated sequences to scan the region of interest of the tissue sample with a specific lateral resolution.

### Construction of elemental images

An advanced spectrum treatment was developed to rapidly extract the intensity for each measurement site and for each species of interest. This algorithm can be applied either in real time, during the mapping scan, or afterward. A single emission line was selected for each element of interest, and the algorithm defined a baseline fit using a polynomial function and subtracted it from the emission signal. Emission lines were selected based on two criteria: each selected line was required to be the strongest line in the probed range and to be unaffected by any possible interference from other lines. To determine the intensity signals for the elements of interest, the algorithm required less than 1 ms, suggesting that a full map (~30,000 spectra) can be processed in less than 30 seconds. A 2D matrix was then provided for each species. Each cell of this matrix represented the intensity signal from a point on the sample surface for the given element and could then be displayed as an image using a false-color scale. The selected emission lines and associated wavelengths are summarized in Supplementary Table I. To measure the strongest lines associated with Gd ions in the UV-visible region, the central wavelength of the spectrometer was set to 333 nm. The spectral range covered in this case, 315 to 350 nm, also allowed the detection of lines originating from Na and Ca.

### Inductively coupled plasma optical emission spectrometry (ICP-OES) analysis

The samples were heated at 550°, and dispersed in 67% HNO_3_ before sonication. Subsequently, the samples were diluted with a 5% HNO_3_ (w/w) matrix to adjust the volume to 20 mL; the samples were then filtered (0.22 μm) and analyzed by ICP-OES (Vista MPX, Varian, France) to determine the elemental content with a precision of 5%. For calibration of the ICP-OES, single-element standard solutions were prepared by successive dilution in a 5% HNO_3_ (w/w) matrix from a 1000-ppm Gd standard acquired from SCP Science.

### 3D elemental renderings

All the 2D sequences were analyzed consecutively, ensuring that no significant angular and/or spatial deviation was present. Elemental images, which were extracted from spectra as described above, were then processed using ImageJ software (NIH, Bethesda, MD, www.nih.gov). Images were contrasted only in a linear manner, slightly smoothed (using a 0.5-pixel Gaussian smoothing), and virtually flipped when necessary and then converted from 16-bit to 8-bit images. The interpolation between the subsequent images was computed using the digital compositing software Morpheus Photo Morpher v3.17. The procedure was successfully used for adjacent slices until sufficient interpolation was achieved; finally, z-stacks were computed. The slice-by-slice reconstructions were visualized by means of surface rendering methods using the open-source software package 3D Slicer v4.4. A false-color scale (green for Gd, red for Na and violet for Ca) was used to present a visual result in the form of elemental renderings. The threshold range was split into high and low intensities, leaving a narrow gap to improve the contrast between them. A metallic effect was also applied, and the image was captured at the angle considered to maximize the 3D effect.

## Additional Information

**How to cite this article**: Gimenez, Y. *et al*. 3D Imaging of Nanoparticle Distribution in Biological Tissue by Laser-Induced Breakdown Spectroscopy. *Sci. Rep.* **6**, 29936; doi: 10.1038/srep29936 (2016).

## Supplementary Material

Supplementary Information
