# Supplementary material for: 3D Imaging of Nanoparticle Distribution in Biological Tissue by Laser-Induced Breakdown Spectroscopy
Source: Sci Rep. 2016 Jul 20;6:29936. doi: 10.1038/srep29936 (PMC4951682; doi:10.1038/srep29936)
Supplement: Supplementary Information [file srep29936-s1.doc]

Manuscript “3D Imaging of Nanoparticle Distribution in Biological Tissue by Laser-Induced Breakdown Spectroscopy” by Y. Gimenez *et al.*

***Supplementary Figure 1***


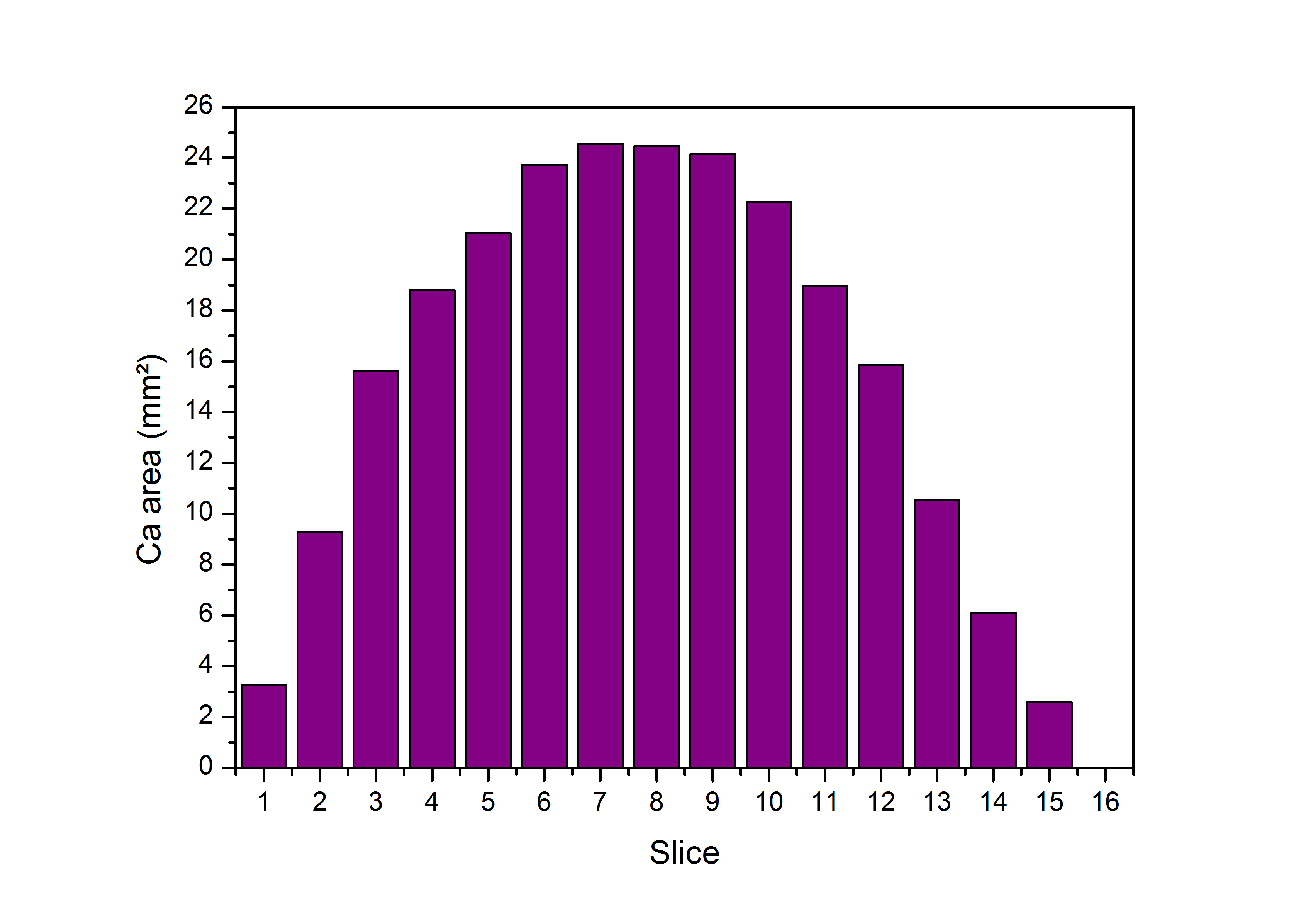


Figure S1: Area of biological tissue estimated for each slices from elemental images of calcium.

Supplementary Table 1

| Element | Specie | Wavelength (nm) |
| --- | --- | --- |
| **Ca** | II | 317.93 |
| **Na** | I | 330.30 |
| **Gd** | II | 342.25 |

Table S1: Lines used for the element of interest.

**Author list**

**Yilbert Gimenez**

Institut Lumière Matière, UMR5306 CNRS

Université Claude Bernard Lyon 1, Campus LyonTech- La Doua

Bâtiment Cléa, 5 rue de la Doua, 69100 Villeurbanne CEDEX, France

yilbert.gimenez@etu.univ-lyon1.fr

**Benoit Busser**

Institut Lumière Matière, UMR5306 CNRS

Université Claude Bernard Lyon 1, Campus LyonTech- La Doua

Bâtiment Cléa, 5 rue de la Doua, 69100 Villeurbanne CEDEX, France

BBusser@chu-grenoble.fr

**Florian Trichard**

Institut Lumière Matière, UMR5306 CNRS

Université Claude Bernard Lyon 1, Campus LyonTech- La Doua

Bâtiment Cléa, 5 rue de la Doua, 69100 Villeurbanne CEDEX, France

florian.trichard@univ-lyon1.fr

**Alexander Kulesza**

Institut Lumière Matière, UMR5306 CNRS

Université Claude Bernard Lyon 1, Campus LyonTech- La Doua

Bâtiment Cléa, 5 rue de la Doua, 69100 Villeurbanne CEDEX, France

alexander.kulesza@univ-lyon1.fr

**Jean-Michel Laurent**

Andor Technology, Springvale Business Park, Belfast BT12 7AL, UK

jm.laurent@andor.com

**Véronique Zaun**

CRITT MATERIAUX ALSACE

19, rue de St Junien C.S. 80023, 67305 Schiltigheim, France

[v.zaun@critt.fr](mailto:v.zaun@critt.fr)

**François Lux**

Institut Lumière Matière, UMR5306 CNRS

Université Claude Bernard Lyon 1, Campus LyonTech- La Doua

Bâtiment Raulin 2 Rue Victor Grignard, 69622 Villeurbanne CEDEX, France

[francois.lux@univ-lyon1.fr](mailto:francois.lux@univ-lyon1.fr)

**Jean-Michel Benoit**

Institut Lumière Matière, UMR5306 CNRS

Université Claude Bernard Lyon 1, Campus LyonTech- La Doua

Bâtiment Brillouin, 6 rue Ada Byron, 69622 Villeurbanne CEDEX, France

[jean-michel.benoit@univ-lyon1.fr](mailto:jean-michel.benoit@univ-lyon1.fr)

**Gérard Panczer**

Institut Lumière Matière, UMR5306 CNRS

Université Claude Bernard Lyon 1, Campus LyonTech- La Doua

Bâtiment Lippmann, 10 rue Ada Byron, 69622 Villeurbanne CEDEX, France

gerard.panczer@univ-lyon1.fr

**Philippe Dugourd**

Institut Lumière Matière, UMR5306 CNRS

Université Claude Bernard Lyon 1, Campus LyonTech- La Doua

Bâtiment Cléa, 5 rue de la Doua, 69100 Villeurbanne CEDEX, France

[philippe.dugourd@univ-lyon1.fr](mailto:philippe.dugourd@univ-lyon1.fr)

**Olivier Tillement**

Institut Lumière Matière, UMR5306 CNRS

Université Claude Bernard Lyon 1, Campus LyonTech- La Doua

Bâtiment Raulin 2 Rue Victor Grignard, 69622 Villeurbanne CEDEX, France

[olivier.tillement@univ-lyon1.fr](mailto:olivier.tillement@univ-lyon1.fr)

**Frédéric Pelascini**

CRITT MATERIAUX ALSACE

19, rue de St Junien C.S. 80023, 67305 Schiltigheim, France

f.pelascini@critt.fr

**Lucie Sancey**

Institut Lumière Matière, UMR5306 CNRS

Université Claude Bernard Lyon 1, Campus LyonTech- La Doua

Bâtiment Raulin 2 Rue Victor Grignard, 69622 Villeurbanne CEDEX, France

[lucie.sancey@univ-lyon1.fr](mailto:lucie.sancey@univ-lyon1.fr)

**Vincent Motto-Ros**

Institut Lumière Matière, UMR5306 CNRS

Université Claude Bernard Lyon 1, Campus LyonTech- La Doua

Bâtiment Cléa, 5 rue de la Doua, 69100 Villeurbanne CEDEX, France

vincent.motto-ros@univ-lyon1.fr
